# Multipoint Left Ventricular Pacing as Alternative Approach in Cases of Biventricular Pacing Failure

**DOI:** 10.3390/jcm14041065

**Published:** 2025-02-07

**Authors:** Christos-Konstantinos Antoniou, Christina Chrysohoou, Panagiota Manolakou, Dimitrios Tsiachris, Athanasios Kordalis, Konstantinos Tsioufis, Konstantinos A. Gatzoulis

**Affiliations:** First Department of Cardiology, National and Kapodistrian University of Athens, Hippokration General Hospital of Athens, 114 Vasilissis Sofias Avenue, 11527 Athens, Greece; ckantoniou@hotmail.gr (C.-K.A.); chrysohoou@usa.net (C.C.); panmanol@gmail.com (P.M.); dtsiachris@yahoo.com (D.T.); akordalis@gmail.com (A.K.); ktsioufis@gmail.com (K.T.)

**Keywords:** cardiac resynchronization therapy, multipoint left ventricular pacing, left ventricular resequencing, conduction system pacing

## Abstract

Cardiac resynchronization therapy (CRT) is a cornerstone in the treatment of dyssynchronous heart failure with reduced ejection fraction. However, the phenomenon of non-response has plagued CRT since its initial application. Notwithstanding issues such as failure to capture the left ventricle, lower-than-required pacing delivery percent, and failure to optimize atrioventricular and interventricular delays, there are patients who fail to exhibit an adequate response to CRT in its classical biventricular pacing (BiVP) form. Several modalities have been proposed as a means to remedy this issue, including pacing the conduction system itself—His or left bundle branch pacing, allowing for intrinsic conduction in some myocardial segments, pacing the left ventricle from multiple points in the coronary sinus (multipoint pacing), or even combining the above (e.g., His/left bundle pacing and BiVP leading to His/left bundle-optimized CRT). In the present review, we present recent evidence for the advantages and disadvantages of each modality and attempt to formulate a pathophysiology and simulation-based strategy to determine the best way forward for delivering CRT in non-responders to BiVP.

## 1. Introduction

Cardiac resynchronization therapy (CRT) remains a cornerstone of dyssynchronous heart failure with reduced ejection fraction (HFrEF) management. Randomized clinical trials [[1,2,3,4]—Table 1] have consistently shown CRT efficacy in alleviating symptoms and prolonging life; even in the absence of defibrillation capacity [5]. Notwithstanding all its limitations; should CRT be used in an evidence-based manner [6]; it will yield significant benefits in patient cardiac function; quality of life; and survival—justifying it being likened to a “minor heart transplantation” dyssynchrony is associated with more than mechanical effects [7]. Changes in the cellular and molecular levels have been detected, including reduced expression of connexins [8] and increased expression of Ca^2+^/calmodulin-dependent protein kinase II and TNFα at the lateral wall, leading to further reduction in cellular synchronization and increased β-adrenergic toxicity, hypertrophy, fibrosis, apoptosis, and arrhythmogenesis [9,10,11,12,13,14]. Furthermore, cellular action potential is prolonged in belatedly activated cells, likely increasing arrhythmogenesis (transmural re-entry) [15,16]. Significantly reduced Ca^2+^ spikes, due to dissociation of T-tubules from the sarcoplasmic reticulum, lead to both reduced power of contraction and increased diastolic calcium levels, again being proarrhythmic [17,18]. Finally, mitochondria themselves appear to spatially dissociate from the sites of greatest energy consumption and exhibit reduced expression of many components necessary for ATP production [7]. Consequently, the reversal of dyssynchrony has effects beyond improved chamber mechanics [19], including better excitation—contraction coupling at the cardiomyocyte level, reduced diastolic calcium levels, correction in connexin expression and localization, as well as restoration of the costamere quasi-organelle, which leads to improved cell-cell and cell-matrix communication and thus adaptability. CRT leads to increases in 31 mitochondrial genes, all associated with components of the electron transport chain and transporters providing necessary molecules for energy production—in short, CRT increases cellular ATP production [20]. Resumption of the adult gene expression pattern has also been reported [21,22], associated with improved work at the cost of slightly reduced efficiency.

The notion of reduced arrhythmogenesis in the context of CRT delivery merits more consideration [38,39,40]. Aside from reducing mechanical stretch and thus mechanosensitive channel activation and ischemia-associated arrhythmogenesis, CRT leads to cellular conformational changes that restore proximity of T-tubules to the sarcomere (potentially altering junctophilin expression), thus reducing the magnitude of calcium spikes necessary to trigger calcium-induced calcium release [41,42]. Indeed, it appears that CRT reduces the incidence of ventricular arrhythmias by 14% (*p* = 0.044) in the primary prophylaxis population while having no effect on secondary prophylaxis patients [43]. A mechanistic interpretation lies in the prevention of disease progression, precluding the formation of potential re-entry circuits (primary prevention patients), while on the other hand imposing continuous epicardial pacing in those with pre-existing arrhythmogenic substrates, increasing the risk for wavefront collision/wavefront break and re-entrant arrhythmias. Super-responders constitute a special subpopulation, with a reduction in arrhythmogenesis of up to 80%, compared to both responders and non-responders [44] (in this context, super-responders were defined as those with a 2-fold or greater improvement in LVEF or a final LVEF ≥ 45% at 12 months post-implantation). Unsurprisingly, the option of downgrading to CRT-P in super-responders has been expressed in the literature [45]; however, the nonzero incidence of malignant arrhythmias in those with ischemic cardiomyopathy and the presence of a defibrillatory lead has mostly rendered this question moot.

All cohorts and registries have consistently reported a percentage of non-response to CRT, delivered through biventricular pacing (BiVP), in the 30% range [46,47,48]. Of the causes associated with this finding, unsuitable coronary vein system anatomy, disease progression, arrhythmia occurrence (e.g., atrial fibrillation), and right ventricular dysfunction (due to intrinsic causes or to the BiVP itself) have been reported, among others [49,50]. Moreover, ischemic cardiomyopathy and non-left bundle branch block (LBBB) QRS complex morphology are well-known predictors of non-response to BiVP-based CRT [51]. Therapeutic inertia is also a major factor since 44% of non-responders were found not to receive any further management [48], with significant effects on hospitalization and survival. Interestingly, only half of those receiving additional management received device reprogramming.

Although dyssynchrony is mainly considered an electrical disease, *mechanical dyssynchrony* depends not only on conduction delays but also on regional excitation–contraction coupling, contractility, and loading conditions, all of which may be affected by CRT [52]. However, there have been conflicting data regarding the role of imaging as a method for predicting and improving response to CRT, pre- and post-implantation. Initially, the PROSPECT trial [53] demonstrated that 12 echocardiography-based parameters available at the time (2008) were unable to predict response to CRT, with areas under the curve not exceeding 0.7 in any case. On the other hand, the STARTER trial [54] demonstrated that pacing the latest *mechanically* activated segment of the LV led to significant improvements in ICD-therapy-free survival (HR 0.64) and in the likelihood of achieving LV resynchronization (72% vs. 48%), which, in turn, was associated with an even more improved therapy-free survival rate (HR 0.49). More sophisticated echocardiographic parameters, such as *mechanical* dispersion (temporal standard deviation between peak longitudinal strain in 17 LV segments) >84 ms at 6 months post-CRT initiation, appeared to predict ventricular arrhythmias and all-cause death [55]. Cardiac magnetic resonance has also been evaluated in the context of predicting/improving response to CRT with similarly encouraging findings—especially given the ability for tissue characterization and thus avoidance of scar areas [56].

Concerning post-implantation optimization, although in specific patients echocardiography-based atrioventricular and interventricular delay programming may lead to improved response [57], a large meta-analysis [58] of more than 4000 patients failed to demonstrate any benefit over empirical programming. This may be partially attributable to the relative contribution of these parameters in each patient, and computational models have been proposed to determine it [59]. Finally, despite the fact that device manufacturers’ proprietary algorithms (based on electrical measurements through the implanted leads) for both automatically adjusting atrioventricular delay (to promote fusion with the endogenous activation wavefront) and suggesting a suitable interventricular delay (to improve dyssynchrony) exist, current evidence for their usefulness is equivocal [60,61]. It appears that a more comprehensive approach is required to deduce the appropriate device programming to achieve a response to CRT.

Even though, when indicated, CRT benefits unequivocally vastly outweigh procedural complication risk, implantation of an additional lead in the coronary sinus (which is nonetheless not necessary anymore in all CRT modalities) does lead to additional complications. A meta-analysis of both randomized clinical trials and clinical registries reported the incidence of CRT (BiVP) complications at 6.9% (registries)—8.1% (clinical trials) [62]. Contrast-induced nephropathy (CIN) is a complication particular to coronary sinus lead implantation due to the need for acquiring a venogram to determine its anatomy and available tributaries. It has been reported in up to 12% of cases and has moreover been associated with an attenuation of CRT benefits among responders—i.e., responders with CIN had lower LVEFs at 6 months (28.5% vs. 35.7%) and lower survival rates at a median follow up of more than 2 years (71% vs. 90%) compared to responders without CIN [63]. A potential explanation may lie in the triggering of type 3/4 cardiorenal syndrome (acute/chronic renocardiac syndrome) with the release of inflammatory cytokines leading to cardiotoxic effects and potentially initiating a vicious cycle [64].

### 1.1. The Concept of Resequencing

In the case of textbook left bundle branch block, cardiac resynchronization therapy can be achieved either by pacing the most belatedly activated portion of the left ventricle (posterior-inferior basal wall segment) through a lead inserted in the coronary sinus (CS), in other words, conventional BiVP or by pacing the left bundle itself—left bundle area pacing—LBAP, the second approach being, not unexpectedly, associated with improved outcomes. In this context, any improvements in QRS complex duration are translatable to improved synchrony and mechanical function. However, when more pronounced and diffuse intraventricular conduction abnormalities are present, often intertwined but not always coinciding with segmental contractility deficits (e.g., an area with affected conduction may alter the timing of an adjacent segment with normal contractility), it is perhaps more prudent to consider the issue of left ventricular dysfunction in the conceptual framework of resequencing rather than resynchronizing [65,66].

More specifically, it is likely that, in such advanced dyssynchrony cases, solely focusing on shortening the QRS segment may necessitate early pacing of areas with few working cardiomyocytes, which affect QRS duration but do not contribute to contractility, thus missing the ultimate goal of improved ventricular function [65,67]. On the other hand, an alternative *sequence* of ventricular activation, tailored on an individual patient basis, may offer improved function, not always associated with the shortest QRS duration. As an example, myocardial segments differ in myosin phosphorylation patterns, allowing fine-tuning of contraction *duration* [68] (e.g., in the papillary muscles). This, and several other concepts, such as cavity three-dimensional shape in order to avoid the formation of vortices [69], are lost when QRS duration is the focus—taken to the extreme this argument suggests that even in cases where QRS duration is not prolonged, yet there are multiple segmental motion abnormalities, the classical activation sequence may not be the optimal sequence any longer—however, there are no robust clinical data to support this—although efforts to use BiVP in HFrEF with normal QRS duration have been reported—with promising results [70].

### 1.2. The Notion of Multipoint Pacing

It follows that, in the case of a ventricle with the pathology described above (multiple segmental conductions and contractility abnormalities, all intertwined), the addition of a second left ventricular activation wavefront may offer more options for properly resequencing the ventricular activation pattern in order to simulate as much as possible the optimal sequence and maximize ventricular function [66]. Consequently, the notion of left ventricular multipoint pacing (MPP) was introduced. In MPP, two different left ventricular pulses may be delivered through a (quadripolar) CS lead. Pacing dipoles and pulses can be categorized into “local”, i.e., between two CS lead poles, and “extended”, i.e., comprising a CS pole and the right ventricular coil. For physiological reasons, no pulses may share the same cathode because the local myocardium will already have been excited by the first pacing pulse, rendering it refractory to the second. In addition to the generation of two activation wavefronts, it has been postulated that pacing at increased pulse energy may lead to anodal stimulation, which in the case of local dipoles, may actually be beneficial inasmuch as it leads to the generation of an additional wavefront [71]—in any case, this phenomenon is thought to occur frequently even in BiVP with CS lead-only dipoles.

Augmentations to both MPP and BiVP have been described; the one most likely to constitute a meaningful addition to their effect is the concept of anticipatory LV pacing (in the form of MPP or BiVP)—leading to conduction system-based right ventricular activation, thus both eliminating iatrogenic dyssynchrony and adding another propagation wavefront [72,73]. Precise programming will require ECG imaging in order to deduce proper programmable delays [74,75]. Taken to the extreme, a “multi-fusion” pacing approach has been described, with a right ventricular pacing pulse being added—regarding outcomes, this approach led on average to QRS duration reductions of almost 40 ms [76]. The ability to take advantage of intrinsic conduction regarding right ventricular activation is in stark contrast to what has been described with LBAP, where the bipolar pacing configuration (potentially capturing both bundles—i.e., distal quasi-His pacing) actually led to worse outcomes than the unipolar one (only capturing the left bundle), attributed to preferential septal activation through the intact right bundle [37].

Due to complex interplays, advanced “digital twin—level” models of cardiac activation latency and contractility will be necessary to assess the global effects of each resequencing option. It is likely that ECG imaging (for conduction) and cardiac magnetic resonance imaging [cMRI—assessing substrate, viability (stress protocols), and contractility] will be sine qua non for acquiring data to be processed and processed, even by means of quantum computing (which excels in optimization problems), in order to derive both the optimal activation sequence and the optimal placement of the CS lead [77,78,79,80].

### 1.3. Clinical Evidence

Before presenting current clinical evidence regarding the role of MPP as a CRT modality in BiVP non-responders, it should be noted that most large trials (Table 1) attempted to optimize MPP using QRS duration as a touchstone, which broadly contradicts the whole framework discussed previously. Furthermore, a clear distinction should be made between studies including patients with LBBB QRS morphology and those enrolling patients with nonspecific intraventricular conduction delays, given that in the latter case MPP effects may be more pronounced than those of conventional BiVP.

MPP in non-responders to BiVP

Compared to optimized BiVP, MPP has been shown to exhibit greater increases regarding $\frac{dP}{dt_{max}}$, external myocardial work, and velocity–time integral at the left ventricular outflow tract ($VTI_{lvot}$) in the acute phase [81,82,83,84]. However, although initial reports were encouraging concerning long-term outcomes and response rates at 12 months (Pappone et al. [85] reported changes in end-systolic LV volume and LV ejection fraction of −25% and +15% with MPP and −18% and +5% with BiVP, respectively), the landmark MultiPoint Pacing Trial [29] only showed noninferiority of MPP response rates compared to BiVP at 3 and 9 months. It should be noted that MPP effects were heavily dependent on programming, and it was through this trial that the notion of wide (>30 mm) anatomical separation between the two LV dipoles and short (5 ms) interventricular delay conferring the most benefit was established—interestingly there was no direct $MPP_{opt.\;anatomy}$ vs. BiVP comparison.

The single-arm HUMVEE clinical trial [31] (employing a cross-over design) uniquely used $VTI_{lvot}$ maximization as the endpoint for optimizing MPP programming. All patients received similarly optimized BiVP for 6 months before switching to optimized MPP for another 6 months. Furthermore, if possible, a local CS dipole with the widest possible anatomical separation between its poles provided the first LV pulse, followed by a second extended second LV pulse configuration with the assumption that this configuration would lead to lateral wall stiffening before apex contraction, facilitating expulsion. There was no attempt to promote intrinsic conduction for right ventricular activation, and the interventricular delay was set to 5 ms, per MPPT findings. Significant improvements were noted regarding 6-min walking distance, NYHA class, and LV functional parameters ($VTI_{lvot}$, stroke volume, ejection fraction, but *not* QRS duration)—although these only persisted when the programmed MPP pacing configuration actually functioned at 12 months—there was a significant 20% of patients with no suitable dipoles at the end of the study. Limitations included potential carry-over effect and inability to assess additional parameter improvement with continuous BiVP *after* the first 6 months. Interestingly, ischemic patients appeared to benefit more from MPP than their nonischemic counterparts, owing to the more diffuse conduction and contraction abnormalities.

As frequently mentioned, the MORE-CRT MPP study [86] failed to demonstrate increased conversion to responders (defined as >15% decrease in LV endsystolic volume) with MPP activation as compared to continued BiVP pacing. Once more, there was evidence for the effects of anatomical dipole separation; however, programming was left to the physicians’ discretion. This was recapitulated in a 2021 meta-analysis [87] confirming the absence of meaningful additional effects of MPP on top of BiVP in randomized trials, with the potential exception of wide dipole anatomical separation, as mentioned multiple times. In stark contrast, a recent (2024) secondary analysis of data from the MORE-CRT MPP cohort [88], focusing on patients with sufficient delivery of CRT (>97% of time, i.e., patients *actually being treated*), showed a statistically significant, including in the multivariate analysis, *increase* in the occurrence of the composite primary endpoint of freedom from cardiac death and heart failure-related hospitalizations and LV endsystolic volume reduction ≥15% (HR 1.55, *p* = 0.04). Notably, of its constituent metrics, both response rates and heart failure-related hospitalizations were significantly reduced in MPP receivers. An additional insight offered by this analysis, useful in constructing a general framework for MPP value, including when pitted against LBAP, is that, contrary to BiVP, its effectiveness remains even in cases with pronounced dispersion of intrinsic LV electrical delay—i.e., when its segments differed significantly in their activation timing, not unexpected based on the generation of an additional wavefront and the capture of more myocardial mass through wide anatomic separation of the LV pulses. In fact, a dispersion of activation timing between LV segments >20 ms [89] or 30 ms [88] has been associated with an advantage of MPP over BiVP regarding response rates (35.5% vs. 17.7%, *p* = 0.0335). Similarly, increased mechanical dispersion (>120 ms) and $\left|GLPS\right|$ (>5%) have been reported to predict additional benefit of MPP over BiVP regarding LVEF (29% vs. 35.7%) [90], although modestly (AUC in the 0.7–0.73 range).

A recent (2024) study [32] reported improved survival in MPP vs. BiVP recipients at 3 years. However, the study design was based on selecting between MPP and BiVP according to improvement in noninvasive hemodynamic parameters upon hospital discharge—thus there was no randomization. Although in theory, this simulates the effects of MPP on BiVP non-responders, potential long-term effects of BiVP on survival could not be assessed in the “non-acute” responders.

To summarize, in theory, MPP offers the potential for more precisely sculpting the choreography of LV activation, adjusted to its current condition (which suggests that it is subject to change with time), ensuring optimal performance [91]. However, MPP is heavily reliant on programming, and this in combination with the absence of an accepted initial programming that can be subsequently improved upon, is a serious practical limitation of MPP, pending the development of advanced simulation models, offering patient-tailored solutions. The concept of interventricular dispersion of activation magnitude is the only currently available predictor for MPP superiority over BiVP.

CS-CRT vs. BiVP

His bundle pacing has always been an attractive alternative pacing modality, ensuring avoidance of pacing-induced cardiomyopathy [92]. Moreover, in cases of LV dyssynchrony attributed to LBBB, conduction system-based CRT (CS-CRT), including both His bundle-based and left bundle-based CRT (HB-CRT and LB-CRT, respectively), has consistently been shown to be superior to conventional BiVP, including when intrinsic activation of the RV is pursued [36,75,93]. In the HOT-CRT trial, HB-CRT showed improvements in the primary efficacy endpoint, namely LV ejection fraction improvement at 6 months (+12.4% vs. +8%, *p* = 0.02). Similar findings have been reported with LB-CRT [94], an approach mitigating several disadvantages of HB-CRT, including high long-term thresholds and difficulties in implantation whilst being non-inferior concerning echocardiographic and functional outcomes [95]. LB-CRT can be safely performed in cases of either HB-CRT failure or the need to revise the His bundle lead [92]. Not only is LB-CRT more often feasible than either HB-CRT and BiVP, but it also leads, compared to the latter, to significantly reduced QRS duration and significantly increased LV ejection fraction, both in absolute and in relative terms. Unsurprisingly, the above effect is driven by an increase in the super-response rate (61.22% vs. 39.22%), given that, should the LB be successfully placed in a patient with LBBB, the chances of super-response are higher. Similar findings were reported in a recent nonrandomized study, with non-responders to BiVP exhibiting a significant decrease in QRS duration and an increase in LV ejection fraction and converting to CRT responders in an impressive 48% [96]. Notably, the presence of LBBB conferred a ninefold higher probability for response to upgrading to LB-CRT. The above have been confirmed in two meta-analyses. The first [97], reported on 11 studies (1 RCT and 10 observational), having enrolled a total of 3141 patients, where LB area pacing was associated with 29% lower mortality and 41% lower risk for HF-related hospitalization. NYHA functional class was also significantly improved with LB area pacing compared to BiVP. Notably, the percentage of patients enrolled in the study with LBBB presence as a criterion was 16%, demonstrating the applicability of LB area pacing even in cases without typical LBBB. The second [98], reporting on 21 studies (4 RCTs and 17 observational) concurred, given that, at a median follow up of 10.1 months, CSP was associated with a 32% reduction in all-cause mortality and a 48% reduction in heart failure-related hospitalization. Additionally, LVEF exhibited significantly increased absolute values in the CSP group (4.26%).

Fusion power

Left bundle pacing-optimized adaptive CRT (LOT-CRT) aims to combine the best of both worlds and enable combined utilization of conduction system endocardial (LB) and epicardial (CS—working myocardium) LV pacing, with the potential to prove advantageous in cases with the coexistence of proximal and distal conduction abnormalities in the LV—the latter not being amenable to correction by LB pacing alone. Indeed, LOT-CRT leads to significantly shorter QRS duration compared to LB-CRT (in the 20 ms range [99,100], with predictors being associated with more advanced disease (such as LV diameter ≥66 mm, LV ejection fraction ≤35% and QRS morphology of LBBB with a duration ≥130 ms). A multicenter trial aiming to assess differences in LV $\frac{dP}{dt_{max}}$ in the acute setting between LB-CRT, BiVP, and LOT-CRT [37] reported that, following optimization in atrioventricular delay, the latter 2 fared better than the former, both in its unipolar and, especially, its bipolar form. Interestingly, although BiVP exhibited (marginally) the greatest increase in LV $\frac{dP}{dt_{max}}$ (26.4% vs. 25.8% vs. LOT-CRT), LOT-CRT was associated with the greatest shortening of QRS duration, a fact pointing to the validity of the assumption that resequencing is a more adequate pursuit than resynchronization and that QRS duration is not its optimal indicator. Finally, LOT-CRT *was* superior to LB-CRT in those with baseline QRS > 171 ms (14.5% higher LV $\frac{dP}{dt_{max}}$, 20.8 ms additional QRS shortening)—thus again in more advanced electrical and structural disease. Notably, due to lead connectology (LB lead connected to RV pacing port, CS lead connected to LV port), LB pacing during LOT-CRT was in the bipolar configuration, associated with anodal stimulation in 54% of cases, leading to reduced effectiveness, as discussed previously. Therefore, the theoretically optimal configuration of (adaptive) unipolar LOT-CRT remains elusive (especially in cases of a defibrillator where unipolar pacing programming is not allowed).

CS-MPP?

No trial has assessed the potential benefits of combining a conduction system (His and LB) and multipoint pacing (CS-MPP), let alone in the presence of additional augmentations, such as allowing for intrinsic RV activation and pursuing a multi-fusion approach. This is surprising, considering that hardware does not differ from that of H/LOT-CRT. Given that up to 4 ventricular stimuli will be administered (His/left bundle, LV1, LV2, RV) and additional wavefronts will be generated either by intrinsic activation or by anodal stimulation, such an approach will absolutely necessitate a digital twin approach, with data from CMR and ECG imaging being integrated in order to determine the optimal configuration for patient-tailored CRT delivery. In a still experimental setting [101,102], optical pacing may allow for almost limitless pacing sequence selections, inasmuch as illuminating an endocardial area of 1 cm^2^, following transfection with genes encoding light-sensitive ion channels, suffices for eliciting ventricular activation. Given that different channels are sensitive to different wavelengths, it follows that an array of colored flashes in the heart may in the future determine the choreography of the left ventricle. Moreover, the insertion of transmural implantable multi-light-emitting diode optical probes has been shown [102], in animal models, to allow for transmural pacing.

**Figure 1:**
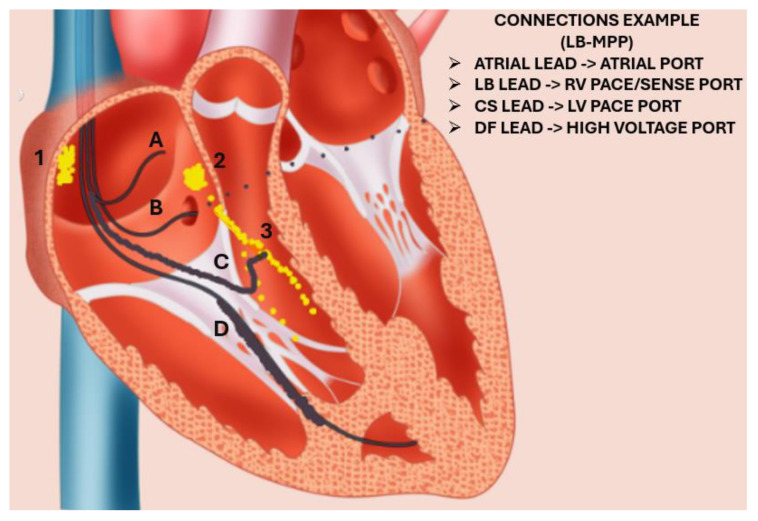
Schematic depiction of lead (drawn in black) position and connection in the case of left bundle branch—optimized MPP. Due to the presence of an ICD, the left bundle branch area pacing pulse must be in the bipolar configuration. Additionally, a DF-1 defibrillation lead is necessary—i.e., split high and low voltage leads, as opposed to a DF-4 (integrated) configuration. 1: Sinoatrial node, 2: Atrioventricular node, 3: Left bundle branch, A: Atrial lead, B: CS lead, C: Left bundle branch pacing lead, D: defibrillation lead at RV apex.

A summary of the relative advantages and disadvantages of the three major CRT methods can be found in Table 2, and a view of the position of various leads in the case of LB-MPP is shown in Figure 1.

### 1.4. Current Perspectives for Non-Responders

Dyssynchrony has profound effects on cardiac function at the mechanical, electrical, cellular, and molecular levels. Thus, CRT is of paramount importance in alleviating these deleterious changes, although plagued by the phenomenon of non-response. The course of CRT has gone from BiVP being the sole available method to the existence of LB-CRT, MPP-CRT, LOT-CRT, and (theoretically) CS-MPP, albeit with various levels of evidence and degrees of certainty. Although rigid evidence is not yet available, physiology and subgroup analyses allow for certain deductions. In cases without response to BiVP, following activation of all available add-ons and optimization of atrioventricular and interventricular delay, one may surmise that for patients with more defined conduction abnormalities and lower intraventricular activation dispersion (<20 ms), H/LB-CRT should be considered, whereas in those with extensive distal disturbances in conduction and contractility, with increased electrical (especially >30 ms) or mechanical (>120 ms) dispersion, the MPP approach should be prioritized. The former approach has the disadvantage of requiring the implantation of an additional lead; however, it also allows for the eventual application of CS-CRT or even CS-MPP if the device can provide them.

## 2. Conclusions

CRT has undergone transformative development during the last decade. From BiVP being the sole means to achieve resynchronization, our arsenal now includes multipoint left ventricular pacing and conduction system pacing. Although large randomized head-to-head comparison clinical trials are lacking, a combinational approach—i.e., CSP on top of BiVP or even MPP—may provide a breakthrough. An improved understanding of the non-mechanical effects of dyssynchrony and the notion of resequencing may provide a theoretical framework to design future approaches. Finally, developments in ECG imaging, CMR, optical pacing, and cardiac function simulation will further advance or completely overhaul our approach to treating dyssynchronous heart failure.

## Figures and Tables

**Table 1:** Available evidence for each CRT method.

Method	Trial/Study (Year of Publication)	Approaches Compared	Findings
BiVP	PATH-CHF [23] (1999)	Univentricular pacing vs. BiVP	Trends for improvement regarding ${\dot{V}}_{O2max}$ and 6-min walking distance
	MUSTIC [24] (2002)	BiVP vs. no pacing (sinus) BiVP vs. Univentricular (patients with Af)	6 min walking +20% ${\dot{V}}_{O2max}$ +10% LVEF +5% Mitral regurgitation improved by 45–50%
	MIRACLE [25] (2002)	BiVP vs. OMT	6 min walking +30 mLVEF +4.6%
	COMPANION [1] (2004)	BiVP+ICD vs. BiVP vs. OMT (2:2:1 randomization)	Primary endpoint: time to death from/hospitalization for any cause BiVP+ICD 0.8 vs. OMT BiVP 0.81 vs. OMT ICD presence had no impact on the endpoint of death from/hospitalization for heart failure.
	MIRACLE-ICD [26] (2003)	BiVP+ICD vs. ICD	BiVP favorably affected quality of life, functional status, and exercise capacity—yet no significant difference in LV function or survival
	CONTAK-CD [27] (2003)	BiVP+ICD vs. ICD	6 min walking +20 m ${\dot{V}}_{O2max}$ +0.8 mL/kg/min LVEF +2.3%
	CARE-HF [2] (2005)	BiVP vs. OMT	Primary endpoint: all-cause death/CV hospitalization HR 0.63 favoring BiVP. Regarding all-cause death specifically HR 0.64 favoring BiVP
	HOBIPACE [28] (2006)	BiVP vs. Univentricular pacing (pacing indicated and LV dysfunction)	Favorable effects of BiVP on LV dimensions, ejection fraction, NT-proBNP levels, and functional status
	RAFT [4] (2010)	BiVP+ICD vs. ICD	Primary endpoint: all-cause death, CV hospitalization HR 0.75 favoring BiVP. Regarding all-cause death HR 0.75 also favors BiVP group
MPP	IRON-MPP (2017)	Registry MPP vs. BiVP	MPP Absolute LVEF increased +4.4% compared to BiVP. Greater benefit if nonischemic cardiomyopathy or QRS > 150 ms
	MPP [29] (2017)	MPP noninferiority to BiVP	MPP non-inferior to BiVP Establishment of anatomical separation of MPP dipoles as part of programming
	MORE-MPP [30] (2019)	MPP in BiVP non-responders	No significant difference—except in those with >97% pacing (subsequent analysis—see text)
	HUMVEE [31] (2022)	Echocardiographically optimized MPP vs. BiVP	Absolute LVEF +4% in MPP Improvement in 6-min walking distance. Significant attrition rate regarding presence of suitable dipoles for MPP may have affected results.
	COMPACT-MPP [32] (2024)	MPP vs. BiVP—nonrandomized, method selected based on noninvasive hemodynamic parameter improvement	Improved survival in MPP recipients at 3 years—however, there was no randomization
CS-CRT	HIS-SYNC [33] (2019)	HB-CRT vs. BiVP	Larger QRS duration shortening, trends toward LVEF improvement
	HIS-ALTERNATIVE [34] (2021)	HB-CRT vs. BiVP	Similar benefits but in the per protocol analysis absolute LVEF +6% in HB-CRT compared to BiVP
	LBBP-RESYNC [35] (2022)	LB-CRT vs. BiVP	Absolute LVEF +5.6% in HB-CRT compared to BiVP. Greater NTproBNP reductionSimilar clinical effects
	HOT-CRT [36] (2023)	HB/LB-CRT vs. BiVP	At 6 months significantly greater relative increase in LVEF in the CSP-CRT group—12.4% vs. 8%—no higher complication rates
	CSPOT [37] (2024)	LBBAP vs. LB-CRT vs. BiVP	Similar hemodynamic effects between LB-CRT and BiVP, superior to those of LBBAP. LB-CRT exhibited the largest QRS duration shortening. LB-CRT superior to LBBAP in those with QRS duration ≥171 ms

**Table 2:** Comparison between currently available methods for CRT delivery.

Feature	BiVP	MPP	CS-CRT
Clearly defined target population	Yes—based on landmark trials	No—a single criterion (electrical dispersion) is currently available for suggesting MPP additional benefit	Indications similar to CRT—there is a trend towards preferential implantation in those with textbook conduction deficits; however, benefits extend to non-specific abnormalities as well
Complexity of implantation	Identical		Simpler, especially for LB-CRT
Maintenance of suitable dipole presence	Most often	Attrition rate of up to 20%	Almost always in LB-CRT Most often in HB-CRT
Simplicity of programming	Average	Complex	Simple
Acute and long-term effects	Both MPP and CP-CRT appear to perform better than BiVP, especially when applied in most suitable cases—i.e., pronounced LV electrical dispersion and LBBB, respectively. H/LOT-CRT has been shown to be superior to conventional BiVP in terms of responder rates and QRS shortening, with 2 meta-analyses suggesting a survival benefit as well.		
Pairing with additional modalities (Multi-fusion, hybrid approaches)	Feasible in all—however, LB-CRT has to be delivered in the bipolar configuration in the presence of a defibrillator, potentially decreasing its efficacy.		

## References

[1] Bristow M.R., Saxon L.A., Boehmer J., Krueger S., Kass D.A., Marco T.D., Carson P., Di Carlo L., De Mets D., White B.G. (2004). Cardiac-Resynchronization Therapy with or without an Implantable Defibrillator in Advanced Chronic Heart Failure. N. Engl. J. Med..

[2] Cleland J.G.F., Daubert J.-C., Erdmann E., Freemantle N., Gras D., Kappenberger L., Tavazzi L. (2005). The Effect of Cardiac Resynchronization on Morbidity and Mortality in Heart Failure. N. Engl. J. Med..

[3] Moss A.J., Hall W.J., Cannom D.S., Klein H., Brown M.W., Daubert J.P., Estes N.A.M., Foster E., Greenberg H., Higgins S.L. (2009). Cardiac-Resynchronization Therapy for the Prevention of Heart-Failure Events. N. Engl. J. Med..

[4] Tang A.S.L., Wells G.A., Talajic M., Arnold M.O., Sheldon R., Connolly S., Hohnloser S.H., Nichol G., Birnie D.H., Sapp J.L. (2010). Cardiac-Resynchronization Therapy for Mild-to-Moderate Heart Failure. N. Engl. J. Med..

[5] Hadwiger M., Dagres N., Haug J., Wolf M., Marschall U., Tijssen J., Katalinic A., Frielitz F.S., Hindricks G. (2022). Survival of patients undergoing cardiac resynchronization therapy with or without defibrillator: The RESET-CRT project. Eur. Heart J..

[6] Glikson M., Nielsen J.C., Kronborg M.B., Michowitz Y., Auricchio A., Barbash I.M., Barrabés J.A., Boriani G., Braunschweig F., Brignole M. (2021). 2021 ESC Guidelines on cardiac pacing and cardiac resynchronization therapy: Developed by the Task Force on cardiac pacing and cardiac resynchronization therapy of the European Society of Cardiology (ESC) With the special contribution of the European Heart Rhythm Association (EHRA). Eur. Heart J..

[7] Cho H., Barth A.S., Tomaselli G.F. (2012). Basic science of cardiac resynchronization therapy: Molecular and electrophysiological mechanisms. Circ. Arrhythmia Electrophysiol..

[8] Spragg D.D., Leclercq C., Loghmani M., Faris O.P., Tunin R.S., DiSilvestre D., McVeigh E.R., Tomaselli G.F., Kass D.A. (2003). Regional alterations in protein expression in the dyssynchronous failing heart. Circulation.

[9] Zhu W.Z., Wang S.Q., Chakir K., Yang D., Zhang T., Brown J.H., Devic E., Kobilka B.K., Cheng H., Xiao R.P. (2003). Linkage of beta1-adrenergic stimulation to apoptotic heart cell death through protein kinase A-independent activation of Ca^2+^/calmodulin kinase II. J. Clin. Investig..

[10] Backs J., Song K., Bezprozvannaya S., Chang S., Olson E.N. (2006). CaM kinase II selectively signals to histone deacetylase 4 during cardiomyocyte hypertrophy. J. Clin. Investig..

[11] Wu X., Zhang T., Bossuyt J., Li X., McKinsey T.A., Dedman J.R., Olson E.N., Chen J., Brown J.H., Bers D.M. (2006). Local InsP3-dependent perinuclear Ca^2+^ signaling in cardiac myocyte excitation-transcription coupling. J. Clin. Investig..

[12] Grueter C.E., Colbran R.J., Anderson M.E. (2007). CaMKII, an emerging molecular driver for calcium homeostasis, arrhythmias, and cardiac dysfunction. J. Mol. Med..

[13] Zhang R., Khoo M.S., Wu Y., Yang Y., Grueter C.E., Ni G., Price E.E., Thiel W., Guatimosim S., Song L.S. (2005). Calmodulin kinase II inhibition protects against structural heart disease. Nat. Med..

[14] Feldman A.M., Combes A., Wagner D., Kadakomi T., Kubota T., Li Y.Y., McTiernan C. (2000). The role of tumor necrosis factor in the pathophysiology of heart failure. J. Am. Coll. Cardiol..

[15] Akar F.G., Rosenbaum D.S. (2003). Transmural electrophysiological heterogeneities underlying arrhythmogenesis in heart failure. Circ. Res..

[16] Beuckelmann D.J., Näbauer M., Erdmann E. (1993). Alterations of K^+^ currents in isolated human ventricular myocytes from patients with terminal heart failure. Circ. Res..

[17] Aiba T., Hesketh G.G., Barth A.S., Liu T., Daya S., Chakir K., Dimaano V.L., Abraham T.P., O’Rourke B., Akar F.G. (2009). Electrophysiological consequences of dyssynchronous heart failure and its restoration by resynchronization therapy. Circulation.

[18] Nishijima Y., Sridhar A., Viatchenko-Karpinski S., Shaw C., Bonagura J.D., Abraham W.T., Joshi M.S., Bauer J.A., Hamlin R.L., Györke S. (2007). Chronic cardiac resynchronization therapy and reverse ventricular remodeling in a model of nonischemic cardiomyopathy. Life Sci..

[19] Antoniou C.-K., Manolakou P., Magkas N., Konstantinou K., Chrysohoou C., Dilaveris P., Gatzoulis K.A., Tousoulis D. (2019). Cardiac Resynchronisation Therapy and Cellular Bioenergetics: Effects Beyond Chamber Mechanics. Eur. Cardiol. Rev..

[20] Agnetti G., Kaludercic N., Kane L.A., Elliott S.T., Guo Y., Chakir K., Samantapudi D., Paolocci N., Tomaselli G.F., Kass D.A. (2010). Modulation of mitochondrial proteome and improved mitochondrial function by biventricular pacing of dyssynchronous failing hearts. Circ. Cardiovasc. Genet..

[21] Barth A.S., Aiba T., Halperin V., DiSilvestre D., Chakir K., Colantuoni C., Tunin R.S., Dimaano V.L., Yu W., Abraham T.P. (2009). Cardiac resynchronization therapy corrects dyssynchrony-induced regional gene expression changes on a genomic level. Circ. Cardiovasc. Genet..

[22] Ståhlberg M., Rullman E., Pernow J., Nakagawa R., Nordin H., Braunschweig F., Ljung K. (2023). Dyssynchrony and resynchronization in heart failure—Effects on regional and global gene expression in a murine pacemaker model. Eur. Heart J. Open.

[23] Auricchio A., Stellbrink C., Sack S., Block M., Vogt J., Bakker P., Mortensen P., Klein H. (1999). The Pacing Therapies for Congestive Heart Failure (PATH-CHF) study: Rationale, design, and endpoints of a prospective randomized multicenter study. Am. J. Cardiol..

[24] Linde C., Leclercq C., Rex S., Garrigue S., Lavergne T., Cazeau S., McKenna W., Fitzgerald M., Deharo J.-C., Alonso C. (2002). Long-term benefits of biventricular pacing in congestive heart failure: Results from the MUltisite STimulation in cardiomyopathy (MUSTIC) study. J. Am. Coll. Cardiol..

[25] Abraham W.T., Fisher W.G., Smith A.L., Delurgio D.B., Leon A.R., Loh E., Kocovic D.Z., Packer M., Clavell A.L., Hayes D.L. (2002). Cardiac Resynchronization in Chronic Heart Failure. N. Engl. J. Med..

[26] Young J.B., Abraham W.T., Smith A.L., Leon A.R., Lieberman R., Wilkoff B., Canby R.C., Schroeder J.S., Liem L.B., Hall S. (2003). Combined Cardiac Resynchronization and Implantable Cardioversion Defibrillation in Advanced Chronic Heart FailureThe MIRACLE ICD Trial. JAMA.

[27] Higgins S.L., Hummel J.D., Niazi I.K., Giudici M.C., Worley S.J., Saxon L.A., Boehmer J.P., Higginbotham M.B., De Marco T., Foster E. (2003). Cardiac resynchronization therapy for the treatment of heart failure in patients with intraventricular conduction delay and malignant ventricular tachyarrhythmias. J. Am. Coll. Cardiol..

[28] Kindermann M., Hennen B., Jung J., Geisel J., Böhm M., Fröhlig G. (2006). Biventricular Versus Conventional Right Ventricular Stimulation for Patients With Standard Pacing Indication and Left Ventricular Dysfunction: The Homburg Biventricular Pacing Evaluation (HOBIPACE). J. Am. Coll. Cardiol..

[29] Niazi I., Baker J., Corbisiero R., Love C., Martin D., Sheppard R., Worley S.J., Varma N., Lee K., Tomassoni G. (2017). Safety and Efficacy of Multipoint Pacing in Cardiac Resynchronization Therapy: The MultiPoint Pacing Trial. JACC Clin. Electrophysiol..

[30] Leclercq C., Burri H., Curnis A., Delnoy P.P., Rinaldi C.A., Sperzel J., Lee K., Calò L., Vicentini A., Concha J.F. (2019). Cardiac resynchronization therapy non-responder to responder conversion rate in the more response to cardiac resynchronization therapy with MultiPoint Pacing (MORE-CRTMPP) study: Results from Phase I. Eur. Heart J..

[31] Antoniou C.K., Dilaveris P., Chrysohoou C., Konstantinou K., Magkas N., Xydis P., Manolakou P., Skiadas I., Gatzoulis K.A., Tousoulis D. (2022). Multipoint left ventricular pacing effects on hemodynamic parameters and functional status: HUMVEE single-arm clinical trial (NCT03189368). Hell. J. Cardiol. HJC = Hell. Kardiol. Ep..

[32] Passafaro F., Rapacciuolo A., Ruocco A., Ammirati G., Crispo S., Pasceri E., Santarpia G., Mauro C., Esposito G., Indolfi C. (2024). COMPArison of Multi-Point Pacing and ConvenTional Cardiac Resynchronization Therapy Through Noninvasive Hemodynamics Measurement: Short- and Long-Term Results of the COMPACT-MPP Study. Am. J. Cardiol..

[33] Upadhyay Gaurav A., Vijayaraman P., Nayak Hemal M., Verma N., Dandamudi G., Sharma Parikshit S., Saleem M., Mandrola J., Genovese D., Tung R. (2019). His Corrective Pacing or Biventricular Pacing for Cardiac Resynchronization in Heart Failure. J. Am. Coll. Cardiol..

[34] Vinther M., Risum N., Svendsen Jesper H., Møgelvang R., Philbert Berit T. (2021). A Randomized Trial of His Pacing Versus Biventricular Pacing in Symptomatic HF Patients With Left Bundle Branch Block (His-Alternative). JACC Clin. Electrophysiol..

[35] Wang Y., Zhu H., Hou X., Wang Z., Zou F., Qian Z., Wei Y., Wang X., Zhang L., Li X. (2022). Randomized Trial of Left Bundle Branch vs Biventricular Pacing for Cardiac Resynchronization Therapy. J. Am. Coll. Cardiol..

[36] Vijayaraman P., Pokharel P., Subzposh F.A., Oren J.W., Storm R.H., Batul S.A., Beer D.A., Hughes G., Leri G., Manganiello M. (2023). His-Purkinje Conduction System Pacing Optimized Trial of Cardiac Resynchronization Therapy vs Biventricular Pacing. JACC Clin. Electrophysiol..

[37] Jastrzębski M., Foley P., Chandrasekaran B., Whinnett Z., Vijayaraman P., Upadhyay G.A., Schaller R.D., Gardas R., Richardson T., Kudlik D.A. (2024). Multicenter Hemodynamic Assessment of the LOT-CRT Strategy: When Does Combining Left Bundle Branch Pacing and Coronary Venous Pacing Enhance Resynchronization?: Primary Results of the CSPOT Study. Circ. Arrhythmia Electrophysiol..

[38] de Vere F., Wijesuriya N., Elliott M.K., Mehta V., Howell S., Bishop M., Strocchi M., Niederer S.A., Rinaldi C.A. (2023). Managing arrhythmia in cardiac resynchronisation therapy. Front. Cardiovasc. Med..

[39] Kies P., Bax J.J., Molhoek S.G., Zeppenfeld K., Bootsma M., Van Erven L., Van der Wall E.E., Shalij M.J. (2005). Long-term follow up of CRT: Effects on arrhythmogenesis: 270 Left ventricular remodeling after cardiac resynchronization therapy reduces inducibility of ventricular tachy-arrhythmias. EP Eur..

[40] Dilaveris P., Giannopoulos G., Synetos A., Aggeli C., Raftopoulos L., Arsenos P., Gatzoulis K., Stefanadis C. (2009). Effect of biventricular pacing on ventricular repolarization and functional indices in patients with heart failure: Lack of association with arrhythmic events. EP Eur..

[41] Lehnart S.E., Wehrens X.H.T. (2022). The role of junctophilin proteins in cellular function. Physiol. Rev..

[42] Heinzel F.R., MacQuaide N., Biesmans L., Sipido K. (2011). Dyssynchrony of Ca^2+^ release from the sarcoplasmic reticulum as subcellular mechanism of cardiac contractile dysfunction. J. Mol. Cell. Cardiol..

[43] Sapp J.L., Parkash R., Wells G.A., Yetisir E., Gardner M.J., Healey J.S., Thibault B., Sterns L.D., Birnie D., Nery P.B. (2017). Cardiac Resynchronization Therapy Reduces Ventricular Arrhythmias in Primary but Not Secondary Prophylactic Implantable Cardioverter Defibrillator Patients. Circ. Arrhythmia Electrophysiol..

[44] García-Lunar I., Castro-Urda V., Toquero-Ramos J., Mingo-Santos S., Moñivas-Palomero V., Daniela Mitroi C., Sánchez-García M., Pérez-Pereira E., Delgado H.E., Fernández-Lozano I. (2014). Ventricular Arrhythmias in Super-responders to Cardiac Resynchronization Therapy. Rev. Española De Cardiol. (Engl. Ed.).

[45] Fang F., Yu C.M. (2014). Shall CRT-D be downgraded to CRT-P in super-responders of cardiac resynchronization therapy?. Rev. Esp. De Cardiol. (Engl. Ed.).

[46] Díaz-Infante E., Mont L., Leal J., García-Bolao I., Fernández-Lozano I., Hernández-Madrid A., Pérez-Castellano N., Sitges M., Pavón-Jiménez R., Barba J. (2005). Predictors of lack of response to resynchronization therapy. Am. J. Cardiol..

[47] Yu C.M., Bleeker G.B., Fung J.W., Schalij M.J., Zhang Q., van der Wall E.E., Chan Y.S., Kong S.L., Bax J.J. (2005). Left ventricular reverse remodeling but not clinical improvement predicts long-term survival after cardiac resynchronization therapy. Circulation.

[48] Varma N., Boehmer J., Bhargava K., Yoo D., Leonelli F., Costanzo M., Saxena A., Sun L., Gold M.R., Singh J. (2019). Evaluation, Management, and Outcomes of Patients Poorly Responsive to Cardiac Resynchronization Device Therapy. J. Am. Coll. Cardiol..

[49] Wouters P.C., Vernooy K., Cramer M.J., Prinzen F.W., Meine M. (2021). Optimizing lead placement for pacing in dyssynchronous heart failure: The patient in the lead. Heart Rhythm.

[50] Sieniewicz B.J., Gould J., Porter B., Sidhu B.S., Teall T., Webb J., Carr-White G., Rinaldi C.A. (2019). Understanding non-response to cardiac resynchronisation therapy: Common problems and potential solutions. Heart Fail. Rev..

[51] Rickard J., Michtalik H., Sharma R., Berger Z., Iyoha E., Green A.R., Haq N., Robinson K.A. (2016). Predictors of response to cardiac resynchronization therapy: A systematic review. Int. J. Cardiol..

[52] Spartalis M., Tzatzaki E., Spartalis E., Damaskos C., Athanasiou A., Livanis E., Voudris V. (2017). The Role of Echocardiography in the Optimization of Cardiac Resynchronization Therapy: Current Evidence and Future Perspectives. Open Cardiovasc. Med. J..

[53] Chung E.S., Leon A.R., Tavazzi L., Sun J.P., Nihoyannopoulos P., Merlino J., Abraham W.T., Ghio S., Leclercq C., Bax J.J. (2008). Results of the Predictors of Response to CRT (PROSPECT) trial. Circulation.

[54] Adelstein E., Alam M.B., Schwartzman D., Jain S., Marek J., Gorcsan J., Saba S. (2014). Effect of echocardiography-guided left ventricular lead placement for cardiac resynchronization therapy on mortality and risk of defibrillator therapy for ventricular arrhythmias in heart failure patients (from the Speckle Tracking Assisted Resynchronization Therapy for Electrode Region [STARTER] trial). Am. J. Cardiol..

[55] van der Bijl P., Khidir M.J.H., Leung M., Yilmaz D., Mertens B., Ajmone Marsan N., Delgado V., Bax J.J. (2018). Reduced left ventricular mechanical dispersion at 6 months follow-up after cardiac resynchronization therapy is associated with superior long-term outcome. Heart Rhythm.

[56] Bazoukis G., Hui J.M.H., Lee Y.H.A., Chou O.H.I., Sfairopoulos D., Vlachos K., Saplaouras A., Letsas K.P., Efremidis M., Tse G. (2022). The role of cardiac magnetic resonance in identifying appropriate candidates for cardiac resynchronization therapy—A systematic review of the literature. Heart Fail Rev..

[57] Waggoner A.D., de las Fuentes L., Davila-Roman V.G. (2008). Doppler echocardiographic methods for optimization of the atrioventricular delay during cardiac resynchronization therapy. Echocardiography.

[58] Auger D., Hoke U., Bax J.J., Boersma E., Delgado V. (2013). Effect of atrioventricular and ventriculoventricular delay optimization on clinical and echocardiographic outcomes of patients treated with cardiac resynchronization therapy: A meta-analysis. Am. Heart J..

[59] Jones S., Lumens J., Sohaib S.M.A., Finegold J.A., Kanagaratnam P., Tanner M., Duncan E., Moore P., Leyva F., Frenneaux M. (2017). Cardiac resynchronization therapy: Mechanisms of action and scope for further improvement in cardiac function. Europace.

[60] Ellenbogen K.A., Gold M.R., Meyer T.E., Fernndez Lozano I., Mittal S., Waggoner A.D., Lemke B., Singh J.P., Spinale F.G., Van Eyk J.E. (2010). Primary Results From the SmartDelay Determined AV Optimization: A Comparison to Other AV Delay Methods Used in Cardiac Resynchronization Therapy (SMART-AV) Trial. Circulation.

[61] Starling R.C., Krum H., Bril S., Tsintzos S.I., Rogers T., Hudnall J.H., Martin D.O. (2015). Impact of a Novel Adaptive Optimization Algorithm on 30-Day Readmissions: Evidence From the Adaptive CRT Trial. JACC Heart Fail..

[62] Pothineni N.V.K., Gondi S., Cherian T., Kovelamudi S., Schaller R.D., Lakkireddy D., Gopinathannair R., Deshmukh A. (2022). Complications of Cardiac Resynchronization Therapy: Comparison of Safety Outcomes from Real-world Studies and Clinical Trials. J. Innov. Card. Rhythm Manag..

[63] Strisciuglio T., Ammirati G., Pergola V., Imparato L., Carella C., Koci E., Chiappetti R., Abbate F.G., La Fazia V.M., Viggiano A. (2019). Contrast-induced nephropathy after cardiac resynchronization therapy implant impairs the recovery of ejection fraction in responders. ESC Heart Fail..

[64] Bagshaw S.M., Hoste E.A., Braam B., Briguori C., Kellum J.A., McCullough P.A., Ronco C. (2013). Cardiorenal syndrome type 3: Pathophysiologic and epidemiologic considerations. Contrib. Nephrol..

[65] Engels E.B., Vis A., van Rees B.D., Marcantoni L., Zanon F., Vernooy K., Prinzen F.W. (2018). Improved acute haemodynamic response to cardiac resynchronization therapy using multipoint pacing cannot solely be explained by better resynchronization. J. Electrocardiol..

[66] Antoniou C.K., Xydis P., Konstantinou K., Magkas N., Manolakou P., Dilaveris P., Chrysohoou C., Gatzoulis K.A., Tsioufis C. (2021). Multipoint left ventricular pacing as an addition to cardiac resynchronization therapy: A bridge to the holy grail?. Am. J. Cardiovasc. Dis..

[67] Sohal M., Shetty A., Niederer S., Lee A., Chen Z., Jackson T., Behar J.M., Claridge S., Bostock J., Hyde E. (2015). Mechanistic insights into the benefits of multisite pacing in cardiac resynchronization therapy: The importance of electrical substrate and rate of left ventricular activation. Heart Rhythm.

[68] Stelzer J.E., Patel J.R., Walker J.W., Moss R.L. (2007). Differential roles of cardiac myosin-binding protein C and cardiac troponin I in the myofibrillar force responses to protein kinase A phosphorylation. Circ. Res..

[69] Siciliano M., Migliore F., Badano L., Bertaglia E., Pedrizzetti G., Cavedon S., Zorzi A., Corrado D., Iliceto S., Muraru D. (2017). Cardiac resynchronization therapy by multipoint pacing improves response of left ventricular mechanics and fluid dynamics: A three-dimensional and particle image velocimetry echo study. Europace.

[70] Bleeker G.B., Holman E.R., Steendijk P., Boersma E., van der Wall E.E., Schalij M.J., Bax J.J. (2006). Cardiac resynchronization therapy in patients with a narrow QRS complex. J. Am. Coll. Cardiol..

[71] Abu Sham’a R., Kuperstein R., Barsheshet A., Bar-Lev D., Luria D., Gurevitz O., Bachar S., Eldar M., Feinberg M., Glikson M. (2011). The effects of anodal stimulation on electrocardiogram, left ventricular dyssynchrony, and acute haemodynamics in patients with biventricular pacemakers. EP Eur..

[72] Dilaveris P., Antoniou C.K., Manolakou P., Skiadas I., Konstantinou K., Magkas N., Xydis P., Chrysohoou C., Gatzoulis K., Tousoulis D. (2019). Comparison of left ventricular and biventricular pacing: Rationale and clinical implications. Anatol. J. Cardiol..

[73] Thibault B., Mondésert B., Cadrin-Tourigny J., Dubuc M., Macle L., Khairy P. (2019). Benefits of Multisite/Multipoint Pacing to Improve Cardiac Resynchronization Therapy Response. Card. Electrophysiol. Clin..

[74] Parreira L., Tsyganov A., Artyukhina E., Vernooy K., Tondo C., Adragao P., Ascione C., Carmo P., Carvalho S., Egger M. (2023). Non-invasive three-dimensional electrical activation mapping to predict cardiac resynchronization therapy response: Site of latest left ventricular activation relative to pacing site. Europace.

[75] Ellenbogen K.A., Auricchio A., Burri H., Gold M.R., Leclercq C., Leyva F., Linde C., Jastrzebski M., Prinzen F., Vernooy K. (2023). The evolving state of cardiac resynchronization therapy and conduction system pacing: 25 years of research at EP Europace journal. EP Eur..

[76] Corbisiero R., Mathew A., Bickert C., Muller D. (2021). Multipoint Pacing with Fusion-optimized Cardiac Resynchronization Therapy: Using It All to Narrow QRS Duration. J. Innov. Card. Rhythm Manag..

[77] Jeyaraman N., Jeyaraman M., Yadav S., Ramasubramanian S., Balaji S. (2024). Revolutionizing Healthcare: The Emerging Role of Quantum Computing in Enhancing Medical Technology and Treatment. Cureus.

[78] Solenov D., Brieler J., Scherrer J.F. (2018). The Potential of Quantum Computing and Machine Learning to Advance Clinical Research and Change the Practice of Medicine. Mo. Med..

[79] Koopsen T., Gerrits W., van Osta N., van Loon T., Wouters P., Prinzen F.W., Vernooy K., Delhaas T., Teske A.J., Meine M. (2023). Virtual pacing of a patient’s digital twin to predict left ventricular reverse remodelling after cardiac resynchronization therapy. EP Eur..

[80] Sel K., Osman D., Zare F., Masoumi Shahrbabak S., Brattain L., Hahn J.O., Pappone C., Ćalović Ž., Vicedomini G., Cuko A. (2024). Building Digital Twins for Cardiovascular Health: From Principles to Clinical Impact. J. Am. Heart Assoc..

[81] Pappone C., Ćalović Ž., Vicedomini G., Cuko A., McSpadden L.C., Ryu K., Romano E., Saviano M., Baldi M., Pappone A. (2014). Multipoint left ventricular pacing improves acute hemodynamic response assessed with pressure-volume loops in cardiac resynchronization therapy patients. Heart Rhythm..

[82] Rinaldi C.A., Leclercq C., Kranig W., Kacet S., Betts T., Bordachar P., Gutleben K.J., Shetty A., Donal E., Keel A. (2014). Improvement in acute contractility and hemodynamics with multipoint pacing via a left ventricular quadripolar pacing lead. J. Interv. Card. Electrophysiol. Int. J. Arrhythm. Pacing.

[83] Thibault B., Dubuc M., Khairy P., Guerra P.G., Macle L., Rivard L., Roy D., Talajic M., Karst E., Ryu K. (2013). Acute haemodynamic comparison of multisite and biventricular pacing with a quadripolar left ventricular lead. Europace.

[84] Zanon F., Baracca E., Pastore G., Marcantoni L., Fraccaro C., Lanza D., Picariello C., Aggio S., Roncon L., Dell’Avvocata F. (2015). Multipoint pacing by a left ventricular quadripolar lead improves the acute hemodynamic response to CRT compared with conventional biventricular pacing at any site. Heart Rhythm.

[85] Pappone C., Ćalović Ž., Vicedomini G., Cuko A., McSpadden L.C., Ryu K., Jordan C.D., Romano E., Baldi M., Saviano M. (2015). Improving cardiac resynchronization therapy response with multipoint left ventricular pacing: Twelve-month follow-up study. Heart Rhythm.

[86] Leclercq C., Burri H., Curnis A., Delnoy P.P., Rinaldi C.A., Sperzel J., Lee K., Cohorn C., Thibault B. (2019). Rationale and design of a randomized clinical trial to assess the safety and efficacy of multipoint pacing therapy: MOre REsponse on Cardiac Resynchronization Therapy with MultiPoint Pacing (MORE-CRT MPP-PHASE II). Am. Heart J..

[87] Mehta V.S., Elliott M.K., Sidhu B.S., Gould J., Porter B., Niederer S., Rinaldi C.A. (2021). Multipoint pacing for cardiac resynchronisation therapy in patients with heart failure: A systematic review and meta-analysis. J. Cardiovasc. Electrophysiol..

[88] Leonardo C., Ermenegildo R., Christof K., Amir J., Pedro M., Pascal D., Christelle M., Olivier P., Andrea G., Kwangdeok L. (2024). Multipoint pacing is associated with improved prognosis and cardiac resynchronization therapy response: MORE-CRT MPP randomized study secondary analyses. Europace.

[89] Wisnoskey B.J., Varma N. (2020). Left ventricular paced activation in cardiac resynchronization therapy patients with left bundle branch block and relationship to its electrical substrate. Heart Rhythm. O2.

[90] Zhu M., Chen H., Fulati Z., Liu Y., Su Y., Shu X. (2019). Left ventricular global longitudinal strain and mechanical dispersion predict response to multipoint pacing for cardiac resynchronization therapy. J. Clin. Ultrasound.

[91] Wijesuriya N., Elliott M.K., Mehta V., De Vere F., Strocchi M., Behar J.M., Niederer S.A., Rinaldi C.A. (2023). Pacing interventions in non-responders to cardiac resynchronization therapy. Front. Physiol..

[92] Archontakis S., Sideris K., Laina A., Arsenos P., Paraskevopoulou D., Tyrovola D., Gatzoulis K., Tousoulis D., Tsioufis K., Sideris S. (2022). His bundle pacing: A promising alternative strategy for anti-bradycardic pacing—Report of a single-center experience. Hell. J. Cardiol..

[93] Feng X.-F., Yang L.-C., Zhao Y., Yu Y.-C., Liu B., Li Y.-G. (2022). Effects of adaptive left bundle branch–optimized cardiac resynchronization therapy: A single centre experience. BMC Cardiovasc. Disord..

[94] Chen X., Ye Y., Wang Z., Jin Q., Qiu Z., Wang J., Qin S., Bai J., Wang W., Liang Y. (2022). Cardiac resynchronization therapy via left bundle branch pacing vs. optimized biventricular pacing with adaptive algorithm in heart failure with left bundle branch block: A prospective, multi-centre, observational study. Europace.

[95] Wu S., Su L., Vijayaraman P., Zheng R., Cai M., Xu L., Shi R., Huang Z., Whinnett Z.I., Huang W. (2021). Left Bundle Branch Pacing for Cardiac Resynchronization Therapy: Nonrandomized On-Treatment Comparison With His Bundle Pacing and Biventricular Pacing. Can. J. Cardiol..

[96] Chen X., Jin Q., Qiu Z., Qian C., Liang Y., Wang J., Qin S., Bai J., Wang W., Chen H. (2024). Outcomes of Upgrading to LBBP in CRT Nonresponders: A Prospective, Multicenter, Nonrandomized, Case-Control Study. JACC Clin. Electrophysiol..

[97] Leventopoulos G., Travlos C.K., Anagnostopoulou V., Patrinos P., Papageorgiou A., Perperis A., Gale C.P., Gatzoulis K.A., Davlouros P. (2023). Clinical Outcomes of Left Bundle Branch Area Pacing Compared with Biventricular Pacing in Patients with Heart Failure Requiring Cardiac Resynchronization Therapy: Systematic Review and Meta-Analysis. RCM.

[98] Kim J.A., Kim S.E., Ellenbogen K.A., Vijayaraman P., Chelu M.G. (2023). Clinical outcomes of conduction system pacing versus biventricular pacing for cardiac resynchronization therapy: A systematic review and meta-analysis. J. Cardiovasc. Electrophysiol..

[99] Jastrzębski M., Moskal P., Huybrechts W., Curila K., Sreekumar P., Rademakers L.M., Ponnusamy S.S., Herweg B., Sharma P.S., Bednarek A. (2022). Left bundle branch–optimized cardiac resynchronization therapy (LOT-CRT): Results from an international LBBAP collaborative study group. Heart Rhythm.

[100] Ribes F., Perez-Rosello V., Gunturiz-Beltran C., De La Cruz-Cereceda S., Bellver-Navarro A. (2024). QRS narrowing in patients undergoing cardiac resynchronization therapy: LOT-CRT vs. LBBAP. EP Eur..

[101] Boyle P.M., Williams J.C., Ambrosi C.M., Entcheva E., Trayanova N.A. (2013). A comprehensive multiscale framework for simulating optogenetics in the heart. Nat. Commun..

[102] Zgierski-Johnston C.M., Ayub S., Fernández M.C., Rog-Zielinska E.A., Barz F., Paul O., Kohl P., Ruther P. (2020). Cardiac pacing using transmural multi-LED probes in channelrhodopsin-expressing mouse hearts. Prog. Biophys. Mol. Biol..

